# Supplementary material for: Fast encirclement of an exceptional point for highly efficient and compact chiral mode converters
Source: Nat Commun. 2022 Apr 19;13:2123. doi: 10.1038/s41467-022-29777-5 (PMC9018827; doi:10.1038/s41467-022-29777-5)
Supplement: Supplementary file 1 — Supplementary Information [file 41467_2022_29777_MOESM1_ESM.docx]

Supplementary material for

**Fast Encirclement of an Exceptional Point for Highly Efficient and Compact Chiral Mode Converters**

Xiaoqian Shu^1,†^, Aodong Li^1,†^, Guangwei Hu^2,3,†^, Jian Wang^1^,*, Andrea Alù^3,4^,* and Lin Chen^1,5^,*

^1^Wuhan National Laboratory for Optoelectronics and School of Optical and Electronic Information, Huazhong University of Science and Technology, Wuhan 430074, China

^2^Department of Electrical and Computer Engineering, National University of Singapore, Singapore, Singapore

^3^Photonics Initiative, Advanced Science Research Center, City University of New York, New York, New York 10031, USA

^4^Physics Program, Graduate Center, City University of New York, New York, New York 10016, USA

^5^State Key Laboratory for Mesoscopic Physics, School of Physics, Peking University, Beijing 100871, China

Email: [jwang@mail.hust.edu.cn](mailto:jwang@mail.hust.edu.cn); [aalu@gc.cuny.edu](mailto:aalu@gc.cuny.edu); [chen.lin@mail.hust.edu.cn](mailto:chen.lin@mail.hust.edu.cn).

* Corresponding author.

† These authors contributed equally to this work.

**Supplementary Note 1: Theoretical analysis of Hamiltonian parameter space**

Equation (1) in the main text can be rewritten as

$$\frac{d|\psi\rangle}{d\tau}=i\left[\begin{matrix} \beta/\kappa+2i\gamma/\kappa& 1 \\ 1 & \beta/\kappa\end{matrix}\right]|\psi\rangle$$

where $\tau$ is the evolution time of the system in the parameter space described by$\vec{s}=(\beta/\kappa,\gamma/\kappa)$, and . The evolution rate of the eigenstate $X_{m}$ along the direction $\vec{s}$ can be written as

$\frac{dX_{m}}{ds}=\frac{\partial X_{m}}{\partial p}\cos\theta+\frac{\partial X_{m}}{\partial q}\sin\theta$,

where $p=\beta/\kappa$, $q=\gamma/\kappa$ and $\theta$ denotes the angle between $\vec{s}$ and $\vec{p}$. Provided that $\mathrm{Im}\left(E_{1}\right)\leq\mathrm{Im}\left(E_{2}\right)$, the two eigenvalues can be expressed as

$E_{1}=\left\{\begin{matrix} \kappa(iq-\sqrt{(p+iq)^{2}+1}) p\geq0 \\ \kappa(iq+\sqrt{(p+iq)^{2}+1}) p<0 \end{matrix}\right.$,

$E_{2}=\left\{\begin{matrix} \kappa(iq+\sqrt{(p+iq)^{2}+1}) p\geq0 \\ \kappa(iq-\sqrt{(p+iq)^{2}+1}) p<0 \end{matrix}\right.$,

with the two normalized eigenstates of

$X_{1}=\left\{\begin{matrix} \frac{\sqrt{2}}{2}\left[\sqrt{1-M},-\sqrt{1+M}\right]^{T} p\geq0 \\ \frac{\sqrt{2}}{2}\left[\sqrt{1+M},\sqrt{1-M}\right]^{T} p<0 \end{matrix}\right.$,

$X_{2}=\left\{\begin{matrix} \frac{\sqrt{2}}{2}\left[\sqrt{1+M},\sqrt{1-M}\right]^{T} p\geq0 \\ \frac{\sqrt{2}}{2}\left[\sqrt{1-M},-\sqrt{1+M}\right]^{T} p<0 \end{matrix}\right.$,

where $M=(p+iq)/\sqrt{1+(p+iq)^{2}}$.

Considering that

$\frac{\partial\sqrt{1\pm M}}{\partial p}=\pm\frac{1}{2\sqrt{1\pm M}}\frac{1}{[1+(p+iq)^{2}]^{3/2}}$,

$\frac{\partial\sqrt{1\pm M}}{\partial q}=\pm\frac{1}{2\sqrt{1\pm M}}\frac{i}{[1+(p+iq)^{2}]^{3/2}}$,

which yields $\frac{\partial X_{m}}{\partial q}=i\frac{\partial X_{m}}{\partial p}$, and equation (S2) can be rewritten as

$\frac{dX_{m}}{ds}=\frac{\partial X_{m}}{\partial p}(\cos\theta+i\sin\theta)=\frac{\partial X_{m}}{\partial p}e^{i\theta}$.

Therefore,

,

which does not depend on the direction of $\vec{s}$.


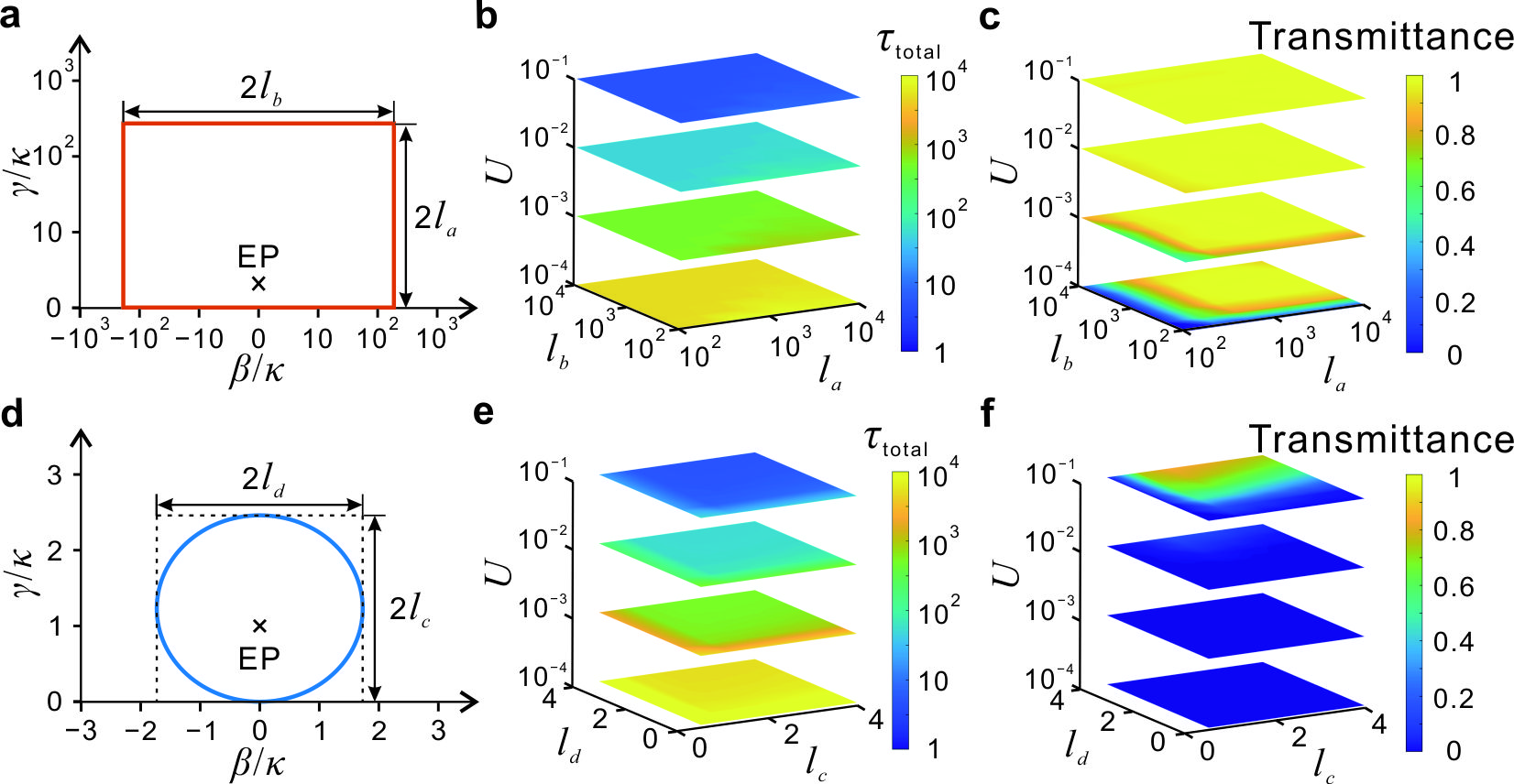


**Supplementary Figure 1. Theoretical analysis with the two encircling loops.** (**a**-**c**) An encircling loop including the Hamiltonian parameter boundaries, and its total evolution time, $\tau_{total}$, and transmittance for different *U*. (**d**-**f**) An encircling loop near the EPs (with a moderate size regime), and its total evolution time, $\tau_{total}$, and transmittance for different *U*. Here, $l_{a}$, $l_{b}$, $l_{c}$ and $l_{d}$ are used to describe the size of encircling loops.

**Supplementary Note 2: CW and ACW loops with initial state** $\left[1,-1\right]^{T}$


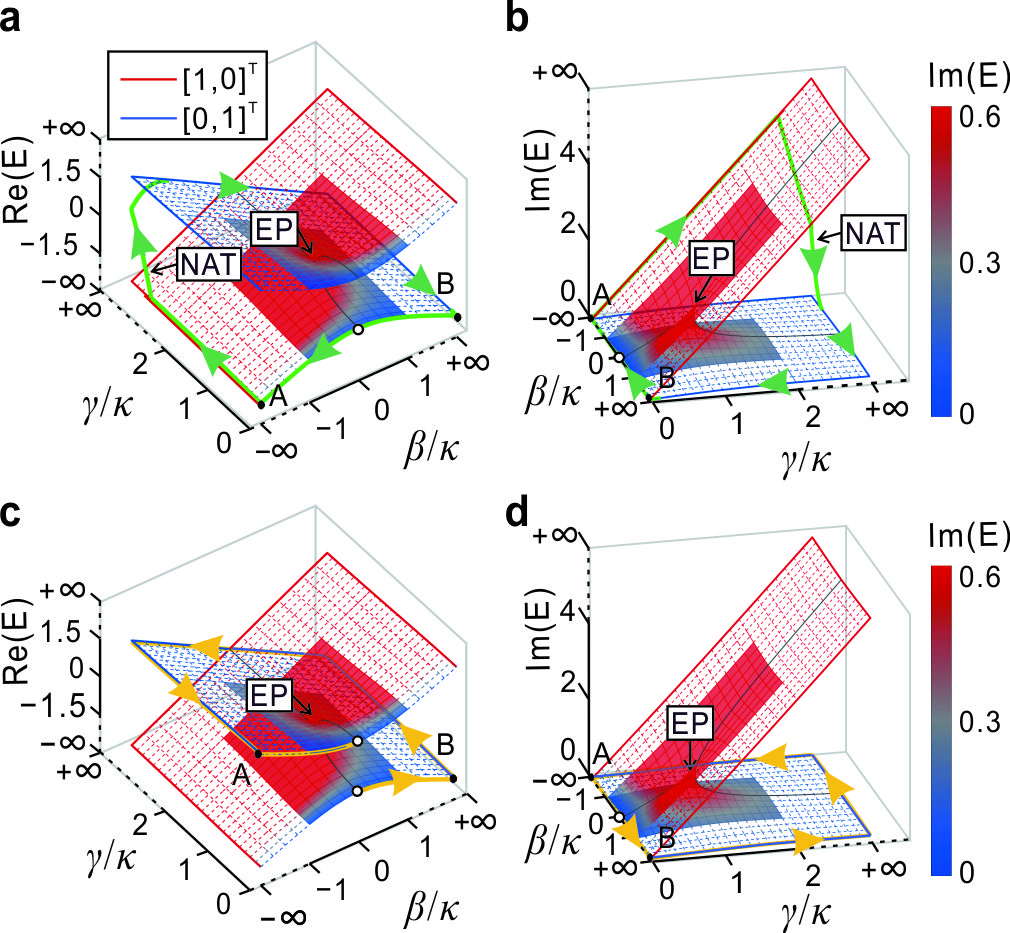


**Supplementary Figure 2. System states evolving on the Riemann surfaces.** (**a**, **b**) CW and (**c**, **d**) ACW loops around an EP in the Riemann surfaces formed by the real part $\mathrm{Re}\left(E\right)$ and the imaginary part $\mathrm{Im}\left(E\right)$ of the energy spectra of $H$ with the initial state of $\left[1,-1\right]^{T}$.

Supplementary Figure 2 shows the dynamic evolution path of the Hamiltonian for the CW (Supplementary Fig. 2a, b) and ACW (Supplementary Fig. 2c, d) loops that include the parameter space boundary denoted by blue and red solid lines between A ($-\infty,0$) and B ($+\infty,0$). The initial state $[1,-1]^{T}$ at the starting point $(\beta/\kappa,\gamma/\kappa)=(0,0)$ is located on the lower sheet of the Riemann surface (Supplementary Fig. 2a, b). For the whole CW loop, the initial state evolves slowly to A, where $X_{2}$ is the dominant eigenstate, and $X_{1}$ is triggered and infinitesimal as adiabatic evolution condition is not strictly fulfilled. $X_{2}$ incurs complete dissipation during the interval between A and B and therefore the lossless eigenstate $X_{1}$ dominates at B, i.e., NAT occurs. The final state returns to $[1,-1]^{T}$ at $\left(0,0\right)$. As for the whole ACW loop (Supplementary Fig. 2c, d), $X_{1}$ is lossless and dominant as the imaginary part of $E_{1}$ is always zero. In contrast, $X_{2}$ is infinitesimal and attenuated when evolving from B to A. The Hamiltonian finally returns to $(0,0)$ with the output state being dominated by $X_{1}=[1,1]^{T}$ on the upper sheet of the Riemann surface.

**Supplementary Note 3: Dynamics of encircling loops**

**
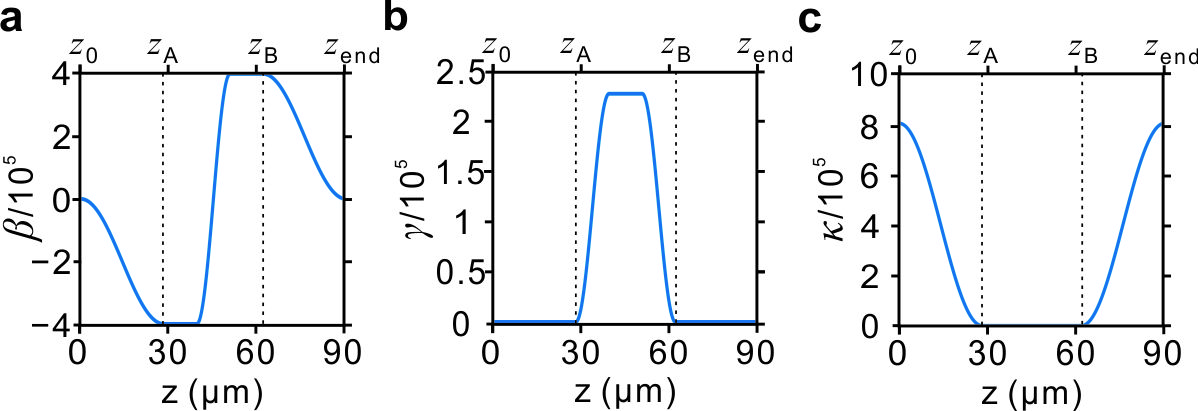
**

**Supplementary Figure 3. Hamiltonian parameters used in the loop**. (**a**) $\beta$, (**b**) $\gamma$, (**c**) $\kappa$ as a function of propagation distance, *z*. In the distance interval of $\left(z_{A},z_{B}\right)$, $\kappa$ is set at an infinitesimal of $10^{3}$.

In order to demonstrate the chiral response as indicated by Fig. 3 in the main text, the selected Hamiltonian parameters are shown in Supplementary Fig. 3. A and B are obtained by letting $\kappa\to0$ and $\gamma=0$, while the path interval between A and B is realized by varied detuning and loss rate. The total propagation distance is assumed to be $Z=90\text{ }\mu m$.

For the whole CW process, $X_{1}$ is always dominant (Supplementary Fig. 4a), and the output state is $\left[1,-1\right]^{T}$(Supplementary Fig. 4b), when the initial state is $\left[1,1\right]^{T}$. For the ACW process, $X_{1}$ is triggered as the adiabatic evolution is not strictly fulfilled. $X_{2}$ incurs high loss in the distance interval of $\left(z_{B},z_{A}\right)$, corresponding to NAT process (Supplementary Fig. 4c). Consequently, the dominant eigenstate shifts to $X_{1}$ after $z_{A}$ and outputs with $\left[1,1\right]^{T}$ (Supplementary Fig. 4d). When the the initial state is $\left[1,-1\right]^{T}$, the final state is $\left[1,-1\right]^{T}$ for the CW process owing to the presence of NAT (Supplementary Fig. 4e, f). For the ACW process, the final output state is $\left[1,1\right]^{T}$(Supplementary Fig. 4g, h). The output state is always dominted by $\left[1,-1\right]^{T}$ for the CW process and $\left[1,1\right]^{T}$ for the ACW process, regardless of the initial input state. It is worth pointing out that high-efficiency chiral mode transmission is guaranteed in principle as the system loss merely occurs during the NAT process, superior over the previous schemes based on the encircling path that is close to EPs. In addition, it can be seen from Supplementary Fig. 4a, c that, the system evolving along the parameter space boundaries will not cause additional crosstalk for the output state. The crosstalk of the output state merely comes from the end of the encircling process, i.e., B to (0, 0) for the CW loop, and A to (0, 0) for the ACW loop.

**
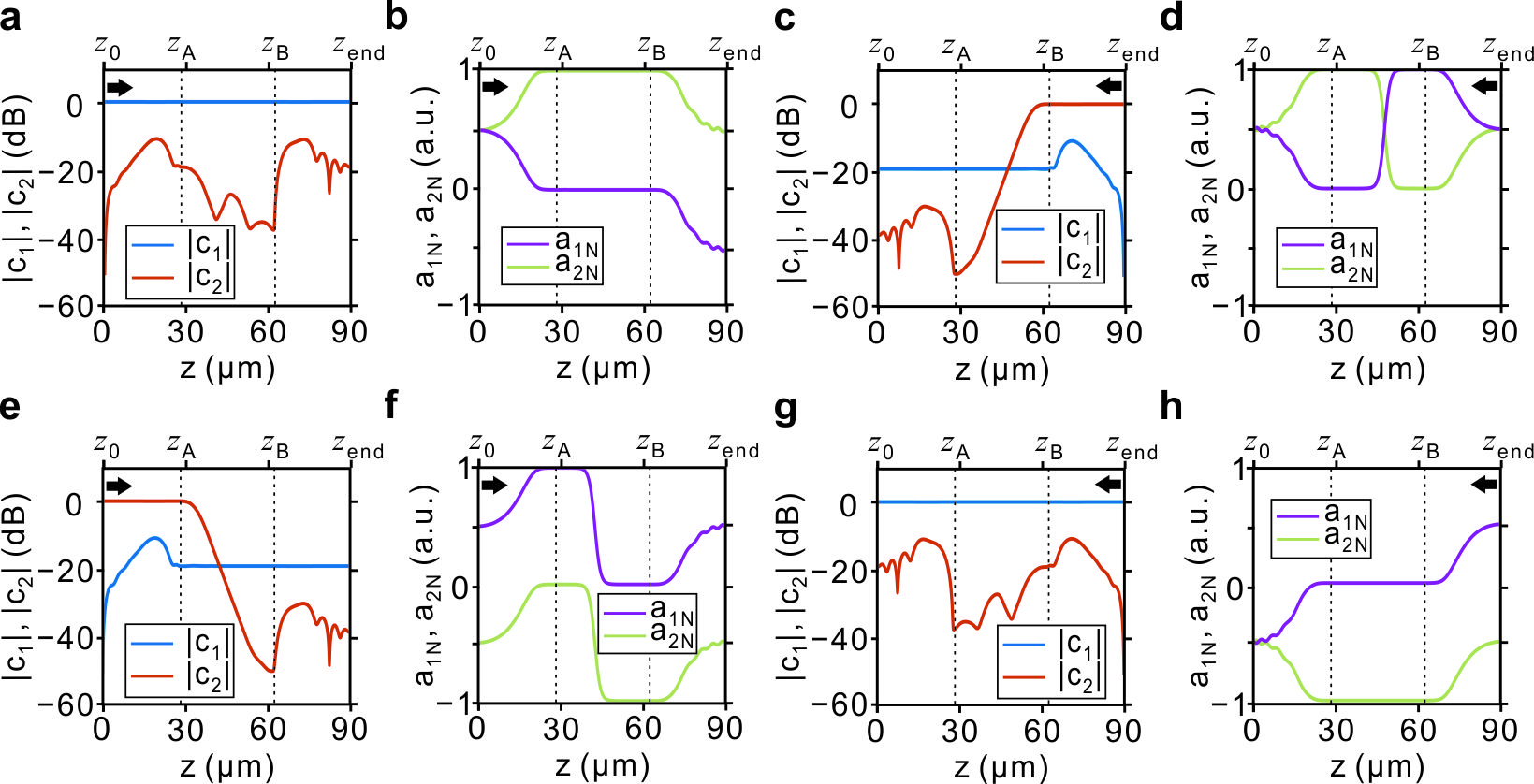
**

**Supplementary Figure 4. Dynamics of the loops including Hamiltonian parameter space boundary**. (**a-d**) The coefficients, $\left|c_{1}\right|$, $\left|c_{2}\right|$, and normalized states, $a_{1N}$, $a_{2N}$ versus propagation distance, *z*, for (a, b) CW and (c, d) ACW loops, when the initial state is $\left[1,1\right]^{T}$. (**e-h**) The coefficients, , $\left|c_{2}\right|$, and normalized states, $a_{1N}$, $a_{2N}$ versus *z* for (e, f) CW and (g, h) ACW loops, when the initial state is $\left[1,-1\right]^{T}$. The normalized states are defined as $a_{1N}=sign(a_{1})\times\left|a_{1}\right|^{2}/(\left|a_{1}\right|^{2}+\left|a_{2}\right|^{2})$ and $a_{2N}=sign(a_{2})\times\left|a_{2}\right|^{2}/(\left|a_{1}\right|^{2}+\left|a_{2}\right|^{2})$, where $sign(a_{1})$ and $sign(a_{2})$ are used to reflect the phase difference at $z_{0}$ and $z_{end}$.

**Supplementary Note 4: Structural parameters**


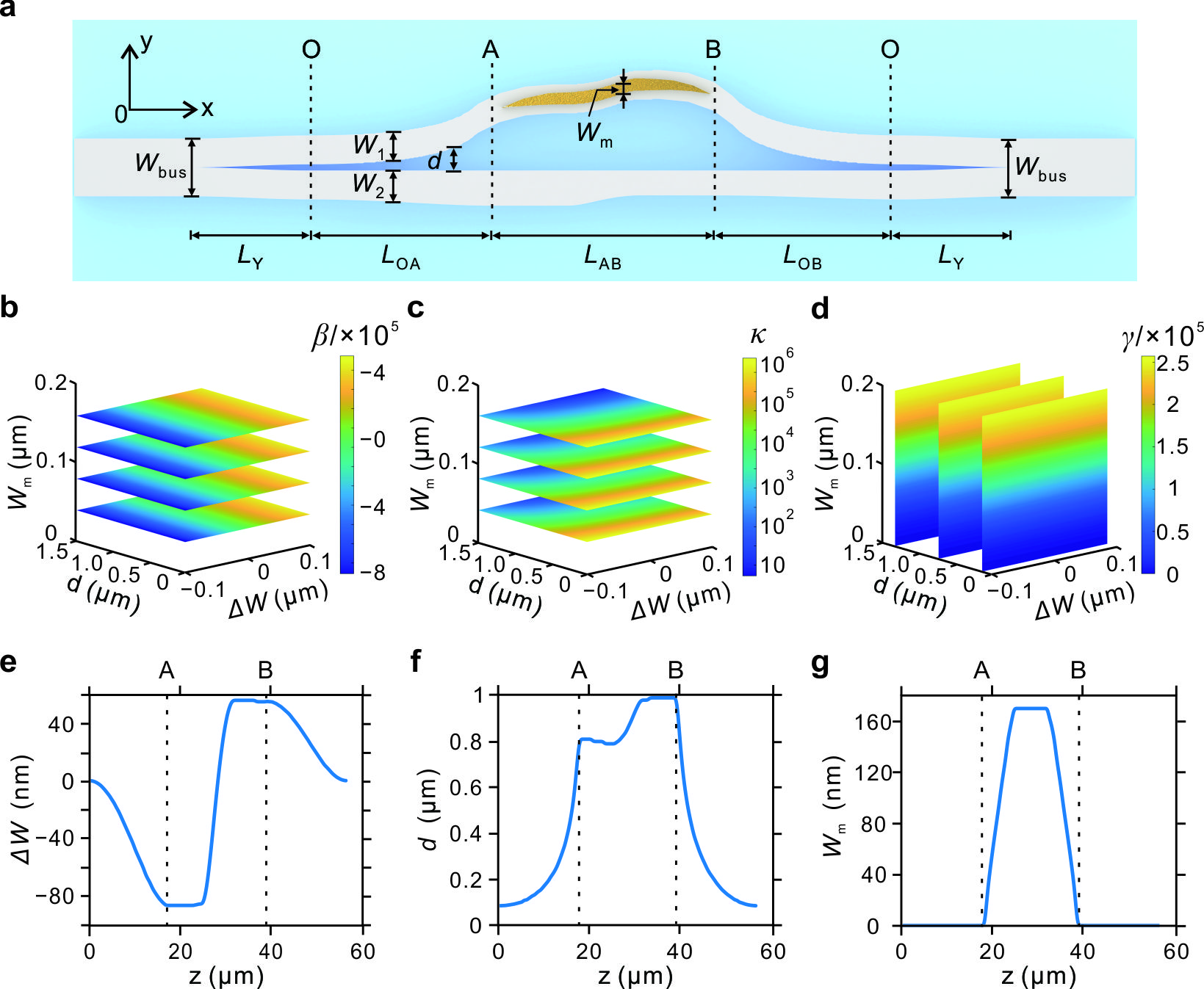


**Supplementary Figure 5. Structural parameters of the coupled waveguides.** (**a**) The top view of double-coupled silicon waveguides. (**b-d**) A part of the mapping database of *β*, *κ*, *γ* with $\Delta W$, $d$ and $W_{m}$ at 1550 nm. (**e-f**) The width difference of the two waveguides, $\Delta W=W_{1}(z)-W_{2}(z)$, the gap distance, $d(z)$, and the width of chromium layer, $W_{m}(z)$, versus z.

Supplementary Figure 5a schematically shows the structural configuration of double-coupled silicon waveguides (DSWs). A part of the mapping database of *β*, *κ*, *γ* with $\Delta W$, $d$, and $W_{m}$ is presented in Supplementary Fig. 5b-d. The dependence of $\Delta W$, $d$ and $W_{m}$ on z is depicted in Supplementary Fig. 5e-g, respectively. The length of the coupled waveguides between the two Y branches is 57 μm with $L_{OA}=L_{OB}=17.8\text{ μm}$ and $L_{AB}=21.4\text{ μm}$. The other geometrical parameters are $W_{1}=0.4\text{ μm}$, $W_{bus}=0.8\text{ μm}$and $L_{Y}=8.2\text{ μm}$.

**Supplementary Note 5: Fabrication details**

Supplementary Figure 6 shows the fabrication process of DSWs samples with a combination of three-step electron-beam lithography (EBL), inductively coupled plasma (ICP) etching, electron-beam evaporation (EBE), and plasma-enhanced chemical vapor deposition (PECVD).

Firstly, an SOI wafer was successively cleaned in ultrasound bath in acetone, isopropyl alcohol and DI water, and then was dried under nitrogen flow. The alignment marks, 20-nm-thick Aurum with 5-nm-thick Chromium as an adhere layer, were fabricated by the first-step EBL, EBE and lift-off process. Photoresist was spin-coated onto the wafer surface and was patterned by EBL, which was followed by development and fixation. The Chromium and Aurum layers were successively deposited by EBE, and the final alignment marks were formed by lift-off process. Secondly, the silicon waveguides and gratings were fabricated by using a second-step EBL and ICP etching. The photoresist was patterned by use of the above-mentioned EBL process, followed by ICP etching to define waveguides and gratings. Thirdly, the Chromium layer on the first waveguides was fabricated by the third-step EBL, EBE and lift-off process. After the photoresist film was spin-coated, the pattern of Chromium is formed by EBL with careful alignment. Subsequently, a 20-nm-thick Chromium layer was deposited using EBE, followed by lift-off process to keep the required Chromium pattern. Finally, a 2-μm-thick SiO_2_ layer is deposited by PECVD, to cover the entire sample for the optical field symmetry and structural protection.


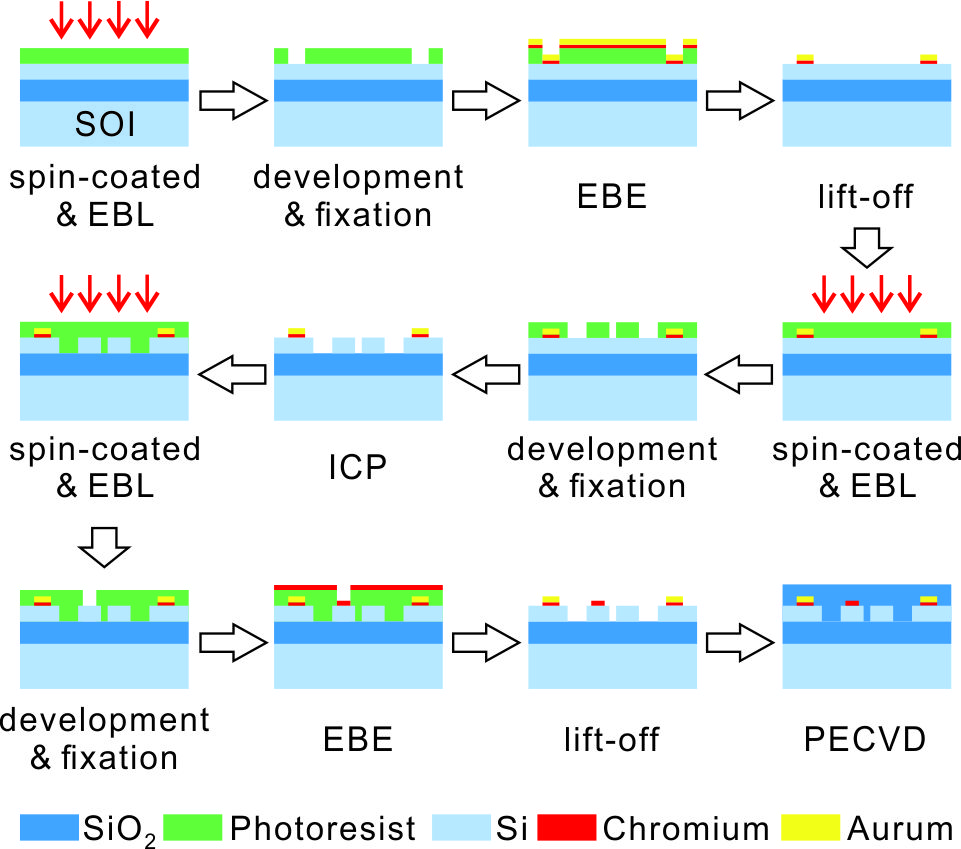


**Supplementary Figure 6. Fabrication process of the samples.**

**Supplementary Note 6: Experimental measurement**


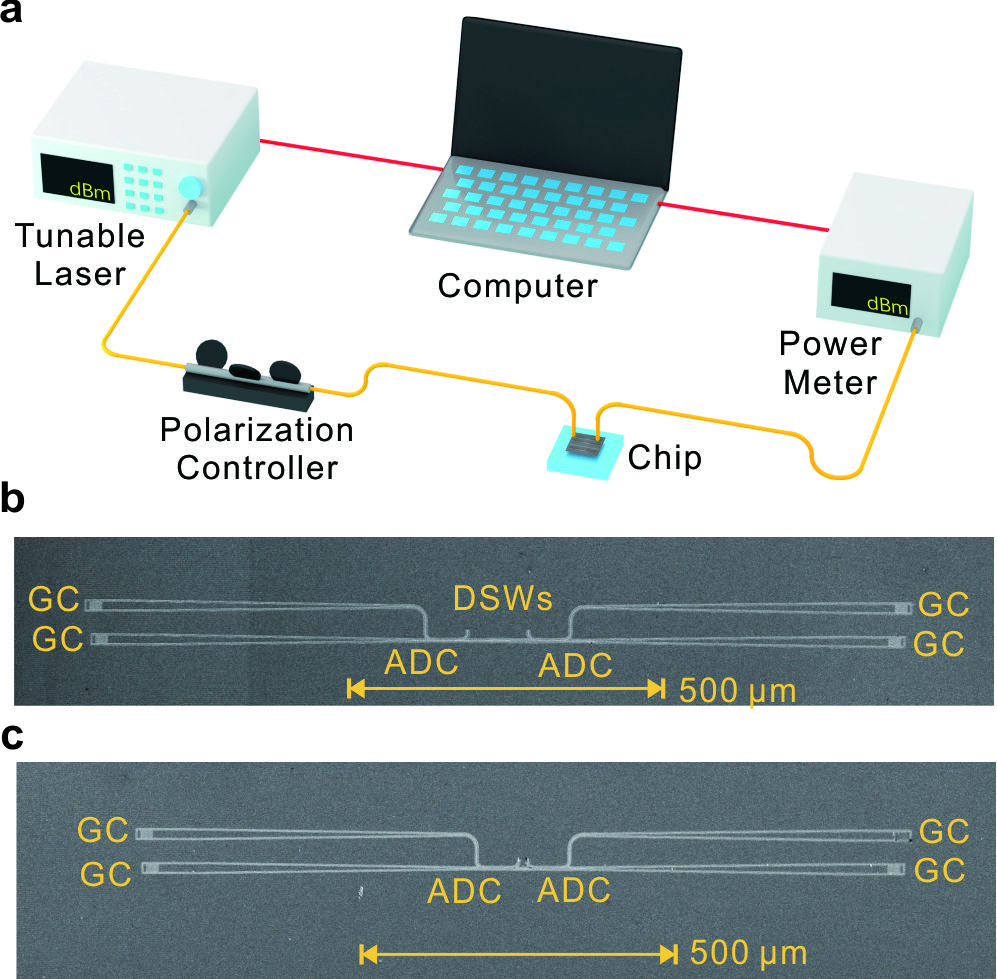


**Supplementary Figure 7. Experimental demonstration.** (**a**) The experimental configuration. (**b**) The SEM image of the fabricated sample consisting of DSWs, GCs, and ADCs. (**c**) The SEM image of the control device without DSWs.

Supplementary Figure 7a shows the experimental configuration for measuring the transmittance of the fabricated DSWs sample. Supplementary Figure 7b, c shows the measured SEM images of the fabricated chip samples. TE_1_ mode separates from the straight bus waveguide and is converted into TE_0_ mode by asymmetrical directional coupler (ADC). The control device without DSWs is used to evaluate the loss arising from ADCs and GCs. The loss differences in Supplementary Figure 7b, c can be used to extract the loss for different TE modes in the DSWs.

The transmittance for TE_0_-TE_1_ exceeds 100% in some wavelengths. This may come from the imperfect fabrication that causes different losses from the GCs and ADCs in the devices with and without DSWs. In our measurement, we have recorded the output power in Supplementary Fig. 7b, c, marked as $P_{out1}$, and $P_{out2}$, as the input power is the same, marked as $P_{in}$. The loss coefficients from the GCs, ADCs, and DSWs are assumed to be $\alpha_{GC}$,$\alpha_{ADC}$, and $\alpha_{DSW}$. We can thus establish two equations for retrieving the transmittance (=): , associated with Supplementary Fig. 7b, and $P_{out2}=P_{in} \cdot e^{-(\alpha_{GC2}+\alpha_{ADC2})}$, associated with Supplementary Fig. 7c. If the total loss coefficient from GCs and ADCs in Supplementary Fig. 7b, , is smaller than that in Supplementary Fig. 7c, $\alpha_{GC2}+\alpha_{ADC2}$, the extracted transmittance can be larger than 100%.

**Supplementary Note 7: Simulated and experimental results with TE_1_ mode input**


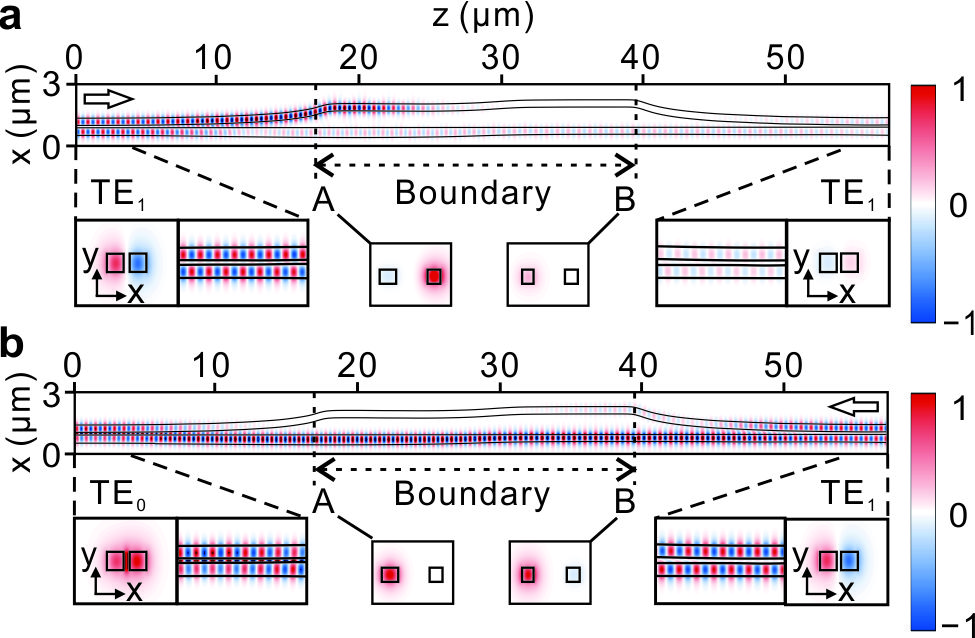


**Supplementary Figure 8. Simulated field distributions.** Simulated field distributions of $E_{x}$ at 1550 nm when the TE_1_ mode inputs from the left port (a) and right port (b), respectively.


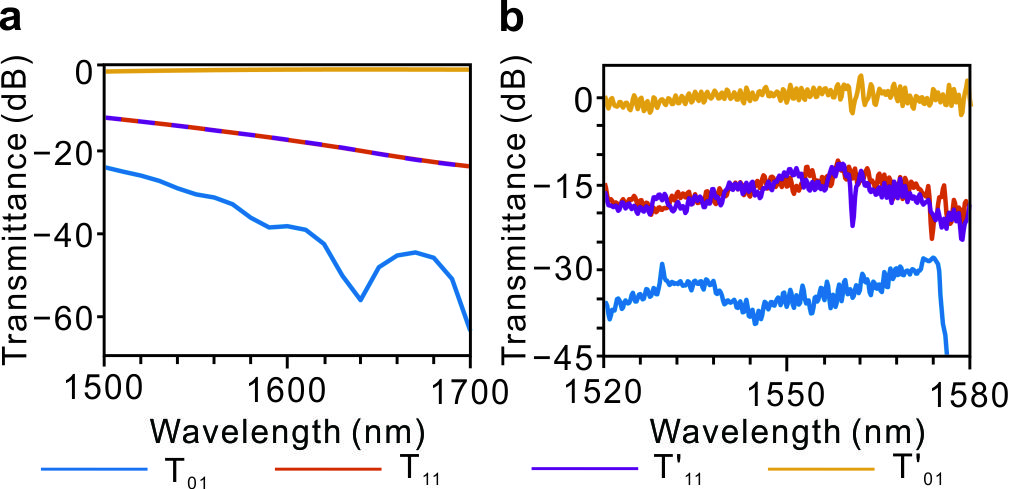


**Supplementary Figure 9. Transmittance spectra.** (**a**) Simulated transmittance spectra for TE_0_ and TE_1_ modes at the output port over the wavelength range of 1500-1700 nm. (**b**) Measured transmittance spectra for TE_0_ and TE_1_ modes at the output port over the wavelength range of 1520-1580 nm.

**Supplementary Note 8: Analytical results with varied device lengths**


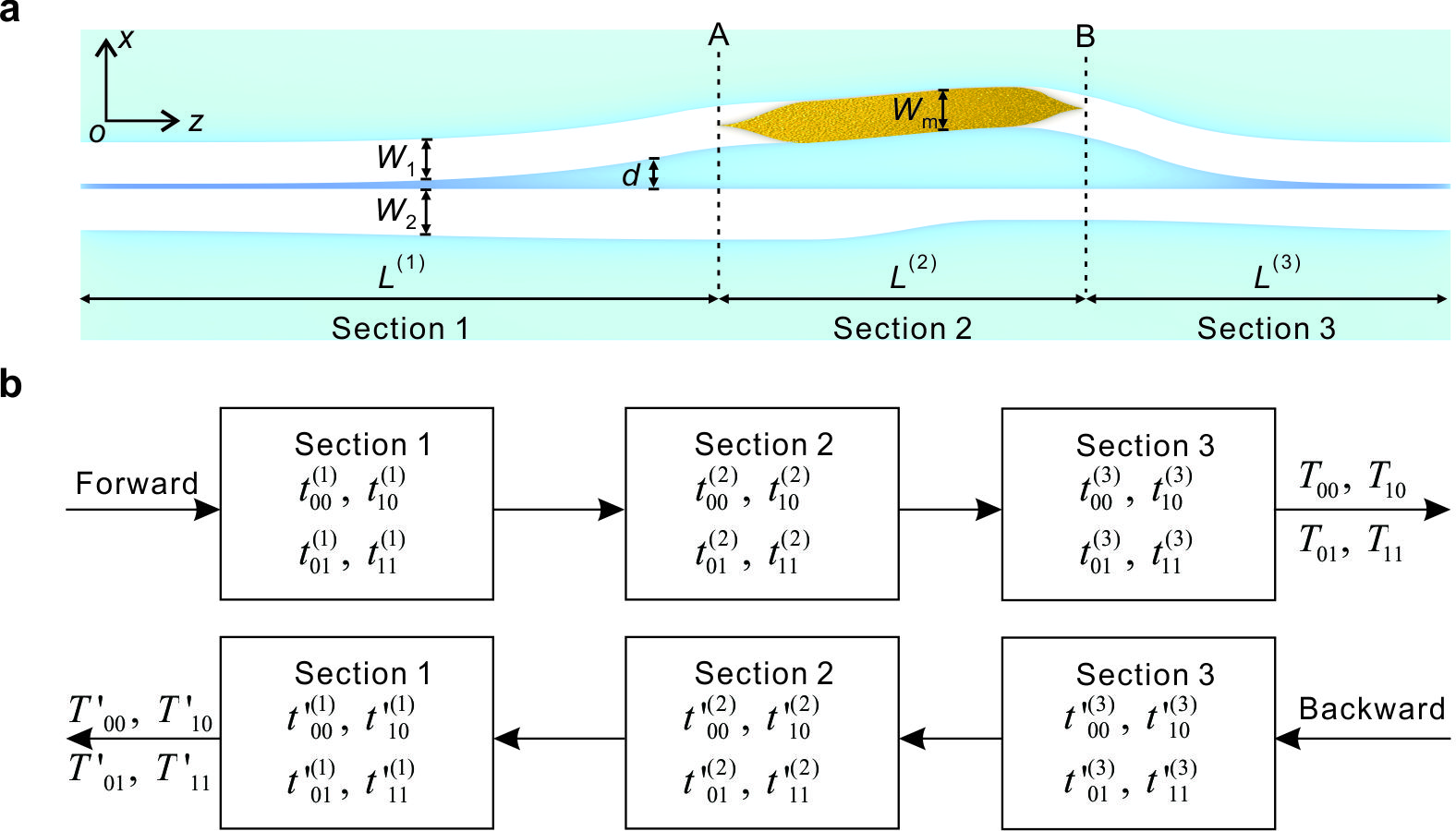


**Supplementary Figure 10. Process of sectional optimization.** (**a**) The top view of double-coupled silicon waveguides used for optimization. (**b**) The transmission efficiency under TE_0_ and TE_1_ modes input for the three sections.

The device is divided into three sections in optimization (Supplementary Fig. 10a). Four geometrical variables are the maximum and minimum gap distances, $d_{\max}^{(N)}$, $d_{\min}^{(N)}$, the maximum width difference of the two waveguides, $\Delta W_{\max}^{(N)}$, and the length, $L^{(N)}$ in each section (the superscript represents the number of the section, N =1, 2, 3). $d$ and $\Delta W$ follow monotone variation in each section. In the section 2, $W_{m}$ is firstly monotonously increased from zero to the maximum value, and then is kept constant. Finally, it is monotonously decreased from the maximum value to zero. It is assumed that the maximum value of $W_{m}$ is no more than $W_{1}$. Without loss of generality, various functions, including $e^{z}$, $z^{4}$, $e^{-z}$, $\ln(z)$, and $\sin z$, have been used to describe the dependence of the geometrical parameters on $z$.

To retrieve $T_{mn}$ ($T'_{mn}$), $t_{uv}^{(N)}$ ($t'_{uv}^{(N)}$) represents the transmission efficiency of TE_u_ mode that outputs from right (left) port when TE_v_ mode inputs from left (right) port in each section (Supplementary Fig. 10b). The transmittance of the entire device can be written as

$$T_{00}=(t_{00}^{(1)}t_{00}^{(2)}+t_{10}^{(1)}t_{01}^{(2)})t_{00}^{(3)}+(t_{00}^{(1)}t_{10}^{(2)}+t_{10}^{(1)}t_{11}^{(2)})t_{01}^{(3)}$$

$$T_{10}=(t_{00}^{(1)}t_{00}^{(2)}+t_{10}^{(1)}t_{01}^{(2)})t_{10}^{(3)}+(t_{00}^{(1)}t_{10}^{(2)}+t_{10}^{(1)}t_{11}^{(2)})t_{11}^{(3)}$$

$$T_{01}=(t_{01}^{(1)}t_{00}^{(2)}+t_{11}^{(1)}t_{01}^{(2)})t_{00}^{(3)}+(t_{01}^{(1)}t_{10}^{(2)}+t_{11}^{(1)}t_{11}^{(2)})t_{01}^{(3)}$$

$$T_{11}=(t_{01}^{(1)}t_{00}^{(2)}+t_{11}^{(1)}t_{01}^{(2)})t_{10}^{(3)}+(t_{01}^{(1)}t_{10}^{(2)}+t_{11}^{(1)}t_{11}^{(2)})t_{11}^{(3)}$$

$$T'_{00}=(t'_{00}^{(3)}t'_{00}^{(2)}+t'_{10}^{(3)}t'_{01}^{(2)})t'_{00}^{(1)}+(t'_{00}^{(3)}t'_{10}^{(2)}+t'_{10}^{(3)}t'_{11}^{(2)})t'_{01}^{(1)}$$

$$T'_{10}=(t'_{00}^{(3)}t'_{00}^{(2)}+t'_{10}^{(3)}t'_{01}^{(2)})t'_{10}^{(1)}+(t'_{00}^{(3)}t'_{10}^{(2)}+t'_{10}^{(3)}t'_{11}^{(2)})t'_{11}^{(1)}$$

$$T'_{01}=(t'_{01}^{(3)}t'_{00}^{(2)}+t'_{11}^{(3)}t'_{01}^{(2)})t'_{00}^{(1)}+(t'_{01}^{(3)}t'_{10}^{(2)}+t'_{11}^{(3)}t'_{11}^{(2)})t'_{01}^{(1)}$$

$$T'_{11}=(t'_{01}^{(3)}t'_{00}^{(2)}+t'_{11}^{(3)}t'_{01}^{(2)})t'_{10}^{(1)}+(t'_{01}^{(3)}t'_{10}^{(2)}+t'_{11}^{(3)}t'_{11}^{(2)})t'_{11}^{(1)}$$

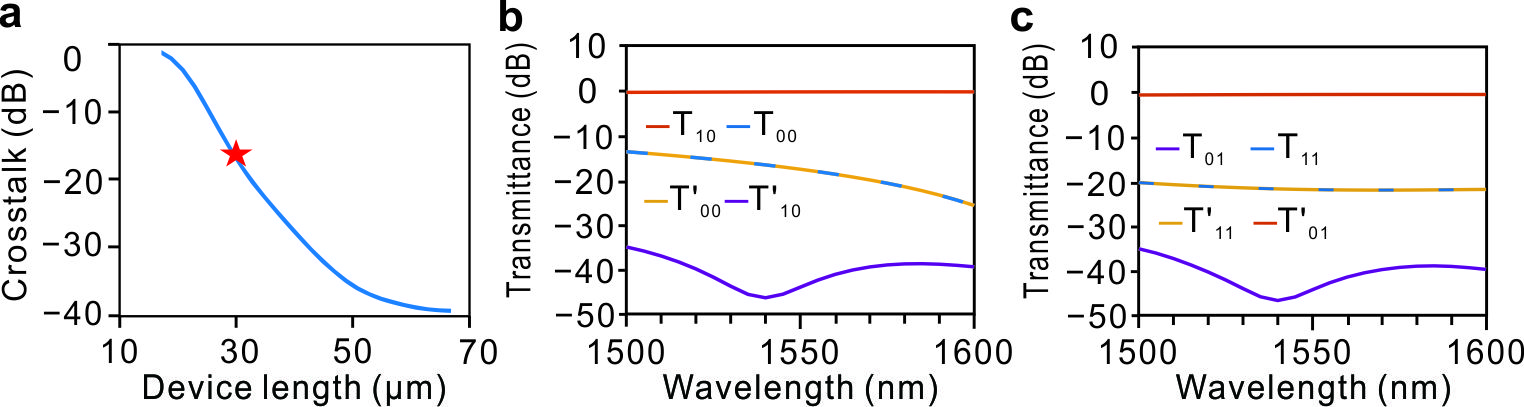


**Supplementary Figure 11. Device performance after sectional optimization.** (**a**) The minimum crosstalk under different device lengths after sectional optimization. The star represents −16 dB crosstalk, associated with the device length of 30 μm. (**b, c**) Simulated transmittance spectra at the output port over the wavelength range of 1500-1600 nm with TE_0_ (b) and TE_1_ (c) modes input, while the entire device of 30 μm length in (a), is simulated with FDTD.

When the efficiency, $T_{01}$, is set to be 96%, the minimum device length is 30 μm with −16 dB minimum crosstalk at 1550 nm (Supplementary Fig. 11a). The other geometrical parameters are $d_{\max}^{(1)}=d_{\min}^{(2)}=0.43\text{ }\mu\text{m}$, $d_{\max}^{(3)}=d_{\max}^{(2)}=0.64\text{ }\mu\text{m}$, $d_{\min}^{(1)}=d_{\min}^{(3)}=0.05\text{ }\mu\text{m}$, $\Delta W_{\max}^{(1)}=0.09\text{ }\mu\text{m}$, $\Delta W_{\max}^{(3)}=0.1\text{ }\mu\text{m}$, $L^{(1)}=14\text{ }\mu\text{m}$, $L^{(2)}=8\text{ }\mu\text{m}$, $L^{(3)}=8\text{ }\mu\text{m}$. The maximum value of $W_{m}$ equals $W_{1}$. The dependence of *d* on z in sections 1 and 3 follows $z^{4}$. The dependence of *d* in section 2 and $\Delta W$ ($W_{m}$) in each section follows $\sin z$. The FDTD simulated transmittance of the entire device in Supplementary Fig. 11b, c is consistent with the analytical results in Supplementary Fig. 11a.

**Supplementary Note 9: Dynamics of a conventional loop**


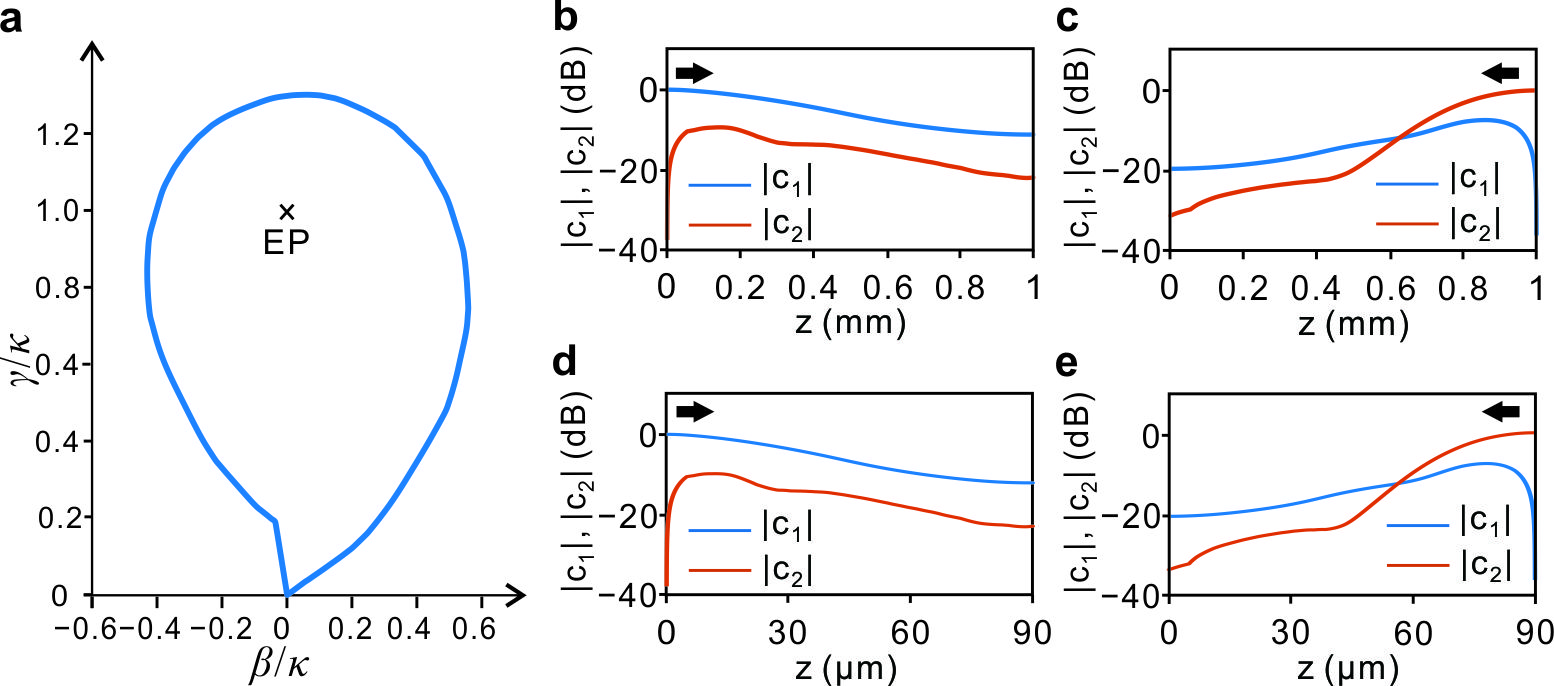


**Supplementary Figure 12. Dynamical loop near the EP.** (**a**) The encircling loop at 1550 nm extracted from Ref. 1. (**b, c**) The coefficients, , $\left|c_{2}\right|$, versus the propagation distance, *z*, for (b) CW and (c) ACW loops, when the initial state is $\left[1,1\right]^{T}$ and $\kappa\approx8500 \text{ m}^{-1}$ (approximately retrieved from the presented structure in Ref. 1). (**d, e**) The coefficients, , $\left|c_{2}\right|$, versus propagation distance, *z*, for (d) CW and (e) ACW loops, when the initial state is $\left[1,1\right]^{T}$ and $\kappa=10^{5} \text{ m}^{-1}$ (assumed value, one order of magnitude larger than the one used in Ref. 1).

The evolution loop used in Ref. 1 is presented in Supplementary Fig. 12a. The extracted *κ* used in the loop is around 8500 m^−1^. We can thus retrieve the Hamiltonian parameters and calculate the dynamics of the loop. The estimated crosstalk is around −10 dB with the length of 1 mm (Supplementary Fig. 12b, c), well consistent with the value shown in Ref. 1. However, if *κ* is increased by one order of magnitude to $10^{5} \text{ m}^{-1}$, the crosstalk is still around −10 dB, but the device length can be shortened to 90 μm (Supplementary Fig. 12d, e).

**Reference**

1. Yoon, J. W. *et al.* Time-asymmetric loop around an exceptional point over the full optical communications band. *Nature* **562**, 86-90, (2018).
